# Update: Influenza Activity — United States and Worldwide, May 19–September 28, 2019, and Composition of the 2020 Southern Hemisphere Influenza Vaccine

**DOI:** 10.15585/mmwr.mm6840a3

**Published:** 2019-10-11

**Authors:** Scott Epperson, C. Todd Davis, Lynnette Brammer, Anwar Isa Abd Elal1, Noreen Ajayi, John Barnes, Alicia P. Budd, Erin Burns, Peter Daly, Vivien G. Dugan, Alicia M. Fry, Yunho Jang, Sara Jo Johnson, Krista Kniss, Rebecca Kondor, Lisa A. Grohskopf, Larisa Gubareva, Angiezel Merced-Morales, Wendy Sessions, James Stevens, David E. Wentworth, Xiyan Xu, Daniel Jernigan

**Affiliations:** 1 Influenza Division, National Center for Immunization and Respiratory Diseases, CDC.

During May 19–September 28, 2019,* low levels of influenza activity were reported in the United States, with cocirculation of influenza A and influenza B viruses. In the Southern Hemisphere seasonal influenza viruses circulated widely, with influenza A(H3) predominating in many regions; however, influenza A(H1N1)pdm09 and influenza B viruses were predominant in some countries. In late September, the World Health Organization (WHO) recommended components for the 2020 Southern Hemisphere influenza vaccine and included an update to the A(H3N2) and B/Victoria-lineage components. Annual influenza vaccination is the best means for preventing influenza illness and its complications, and vaccination before influenza activity increases is optimal. Health care providers should recommend vaccination for all persons aged ≥6 months who do not have contraindications to vaccination (*1*).

## Surveillance Update: United States and Worldwide

**FIGURE 1:**
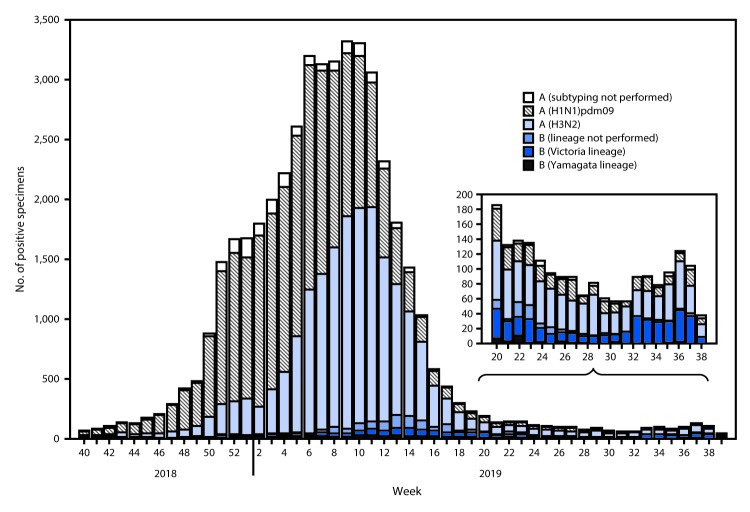
Number of respiratory specimens testing positive for influenza* reported by public health laboratories, by influenza virus type, subtype/lineage, and surveillance week — United States, September 30, 2018–September 28, 2019^† ^<Fig_Large></Fig_Large> * N = 45,619. ^†^ As of October 4, 2019.

The U.S. Influenza Surveillance System^†^ is a collaboration between CDC and federal, state, local, and territorial partners and uses eight data sources, six of which operate year-round, to collect clinical and laboratory information on influenza. During May 19–September 28, 2019 (surveillance weeks 21–39), public health laboratories in the United States tested 7,637 respiratory specimens for influenza viruses; 1,737 (22.7%) were positive (Figure 1), including 1,213 (69.8%) for influenza A viruses and 524 (30.2%) for influenza B viruses. Among the 1,154 seasonal influenza A-positive specimens that were subtyped, 324 (28.1%) were influenza A(H1N1)pdm09, and 830 (71.9%) were influenza A(H3N2). Among the 440 influenza B viruses for which lineage was determined, 413 (93.9%) belonged to the B/Victoria lineage and 27 (6.1%) to the B/Yamagata lineage.

During May 19–September 28, 2019, the weekly percentage of outpatient visits to health care providers for influenza-like illness (ILI) from the U.S. Outpatient Influenza-Like Illness Surveillance Network (ILINet) was below the national baseline, and all regions were below their region-specific baselines. One human infection with a novel influenza A virus^§^ was reported, an influenza A(H1N1) variant virus. This virus had hemagglutinin (HA) and neuraminidase gene segments derived from the seasonal human influenza A(H1N1)pdm09 virus that were likely introduced into swine by a recent reverse zoonosis and were closely related to influenza A(H1N1) viruses now circulating in the U.S. swine population. The percentage of deaths attributed to pneumonia and influenza from CDC’s National Center for Health Statistics Mortality Surveillance System was below the epidemic threshold during this period. Five influenza-associated pediatric deaths occurring during this period were reported to CDC. Additional information on influenza surveillance methods is available at https://www.cdc.gov/flu/weekly/overview.htm, and a full description of U.S. influenza activity over the summer months is available in the influenza surveillance report, FluView (https://www.cdc.gov/flu/weekly/).

The timing of influenza activity and the predominant circulating virus in the Southern Hemisphere during May 19–September 28, 2019 varied by region.^¶^ Influenza A(H3N2) viruses were predominant in most regions; however, influenza A(H1N1)pdm09 and influenza B/Victoria viruses predominated in several countries. Additional information on global influenza virus circulation is available at https://www.who.int/influenza/surveillance_monitoring/updates/en/.

## Genetic and Antigenic Characterization of Influenza Viruses

**FIGURE 2:**
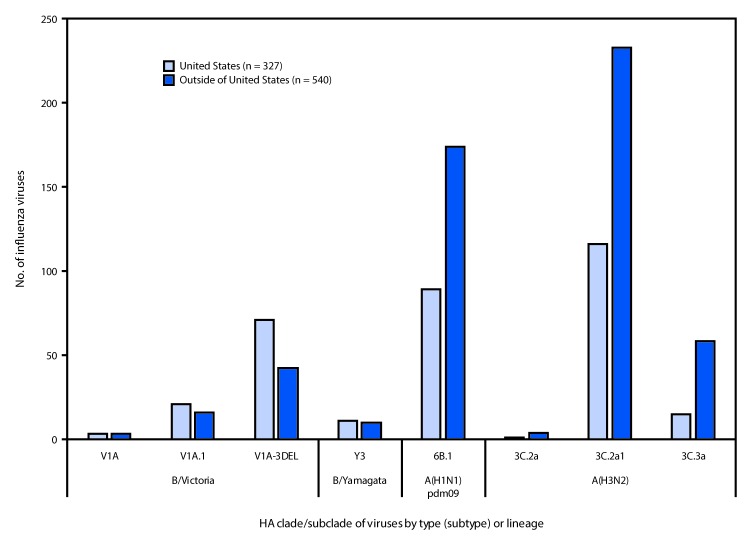
Genetic characterization of U.S. and global viruses collected during May 19–September 28, 2019

CDC genetically characterized 867 influenza viruses submitted by U.S. and international laboratories during May 19–September 28, 2019, including 263 influenza A(H1N1)pdm09 viruses, 427 influenza A(H3N2) viruses, and 177 influenza B viruses. All A(H1N1)pdm09 viruses belonged to genetic subclade 6B.1A. Among 25 antigenically characterized A(H1N1)pdm09 viruses, 96% were similar** to the cell-culture propagated 2019–20 Northern Hemisphere vaccine virus component. The 427 influenza A(H3N2) viruses analyzed belonged to either clades 3C.2a (354; 83%) or 3C.3a (73; 17%) (Figure 2). Multiple subclades within the 3C.2a clade cocirculated with the majority of viruses belonging to subclade 3C.2a1, with regional differences in which subgroup of 3C.2a1 predominated. A(H3N2) viruses with a clade 3C.3a HA, which reemerged last season, continue to circulate in the WHO Region of the Americas. Among the 74 representative A(H3N2) viruses antigenically characterized, 70% were similar to the cell-culture propagated 2019–20 Northern Hemisphere vaccine virus component. Thus, although ferret antisera clearly distinguish antigenic differences between 3C.2a and 3C.3a viruses there is some cross-reactivity.

All 21 of the influenza B/Yamagata lineage viruses analyzed belonged to clade Y3. All seven B/Yamagata lineage viruses antigenically characterized were similar to the cell culture–propagated 2019–20 Northern Hemisphere vaccine virus component. Multiple genetically and antigenically distinct B/Victoria lineage viruses cocirculated. Viruses with a two-amino acid deletion (162–163) in the HA protein belonged to subclade V1A.1, and viruses with a three-amino acid deletion (162–164) in the HA protein belonged to subclade V1A-3Del. Among the 156 influenza B/Victoria lineage viruses analyzed, the HA gene belonged to clade V1A (six viruses; 4%), subclade V1A.1 (37; 24%), or subclade V1A-3Del (113; 72%). Among the 53 B/Victoria lineage viruses antigenically characterized, the V1A.1 viruses were similar to the cell culture–propagated 2019–20 Northern Hemisphere vaccine component. Ferret antisera raised to recent V1A.1 viruses, however, had reduced reactivity with many viruses expressing V1A and V1A-3Del HA proteins indicating some antigenic differences between viruses in the different B/Victoria lineage subclades. Nevertheless, sera from humans vaccinated with a V1A.1 virus cross reacted well with V1A-3Del viruses.

## Antiviral Resistance of Influenza Viruses

CDC tested 812 influenza virus specimens collected during May 19–September 28 from the United States and worldwide for resistance to oseltamivir, peramivir, and zanamivir. All but two of the viruses tested (245 influenza A(H1N1)pdm09 viruses [161 international and 84 U.S. viruses], 406 influenza A(H3N2) viruses [284 international and 122 U.S.], and 161 influenza B viruses [71 international and 90 U.S.]) were susceptible to these influenza antiviral medications. One (0.1%) influenza A(H1N1)pdm09 virus contained the H275Y amino acid substitution in the neuraminidase and exhibited highly reduced inhibition by oseltamivir and peramivir, and one (0.1%) influenza B virus contained the amino acid substitution I221T and exhibited reduced inhibition by the same two neuraminidase inhibitors. Among 824 influenza virus specimens (253 A(H1N1)pdm09, 406 A(H3N2) and 165 type B assessed for susceptibility to baloxavir, one (0.1%) A(H3N2) virus contained amino acid substitution I38L in the polymerase acidic (PA) protein, which was previously associated with at least a threefold decreased baloxavir susceptibility. High levels of resistance to the adamantanes (amantadine and rimantadine) persisted among influenza A(H1N1)pdm09 and influenza A(H3N2) viruses, which is consistent with the current recommendation to avoid use of these medications against influenza. Influenza antiviral recommendations are available at https://www.cdc.gov/flu/professionals/antivirals/links.htm.

## Composition of the 2020 Southern Hemisphere Influenza Vaccine

WHO recommendations for influenza vaccine composition for the Southern Hemisphere 2020 season were made at the WHO Consultation and Information Meeting on the Composition of Influenza Virus Vaccines held September 23–27, 2019, in Geneva, Switzerland.^††^ The recommended components for the 2020 Southern Hemisphere egg-based influenza trivalent vaccines are an A/Brisbane/02/2018 (H1N1)pdm09-like virus, an A/South Australia/34/2019 (H3N2)-like virus, and a B/Washington/02/2019-like virus (B/Victoria lineage). For egg-based quadrivalent vaccines, an additional component, B/Phuket/3073/2013-like virus (B/Yamagata lineage), is recommended. It was recommended that the A(H3N2) component of non–egg-based vaccines be a cell-propagated A/Iowa/60/2018-like virus.

## Discussion

From May to September 2019, influenza activity remained low in the United States, as is typical for that time of year. Influenza A and B viruses cocirculated throughout the summer months with influenza A(H3N2) viruses predominating overall and influenza B/Victoria, subclade V1A-3Del, viruses the most common influenza B virus reported by public health laboratories. Influenza A and B viruses also circulated widely in the Southern Hemisphere with the predominant virus varying by region and country. It is too early in the season to know which viruses will circulate in the United States later this fall and winter or how severe the season might be; however, regardless of what is circulating, the best protection against influenza is an influenza vaccination. Influenza vaccination has been shown to reduce the risk for influenza illness associated with outpatient health care visits and hospitalizations and reduces the risk for serious influenza outcomes that can result in hospitalization or death. CDC recommends that all persons aged 6 months and older who do not have contraindications get vaccinated, but vaccination is especially important for persons at high risk for serious influenza-associated complications, including persons aged ≥65 years, children aged <5 years, pregnant women, and persons with certain underlying medical conditions.

In late September, WHO issued its recommendations for the 2020 Southern Hemisphere influenza vaccine. Compared with the composition of the 2019–20 Northern Hemisphere influenza vaccine formulation, these recommendations reflect changes to the A(H3N2) and B/Victoria-lineage components. The update for the B/Victoria-lineage component reflects the global spread and increase of V1A-3Del viruses, which had reduced reactivity to ferret antisera raised to V1A.1 viruses used in 2019–20 Northern Hemisphere vaccines. Apart from North and South America, the majority of A(H3N2) viruses circulating elsewhere globally belonged to subclade 3C.2a1 and were antigenically different from the Northern Hemisphere 3C.3a vaccine component, leading to a change in the A(H3N2) component to a 3C.2a1 subclade virus for the Southern Hemisphere. These recommendations were made specifically for the Southern Hemisphere using many factors, including evolutionary approaches to forecast specific subgroups likely to circulate 6 months into the future, determining which candidate vaccine viruses induce immunity that blocks the largest variety of viruses and which viruses escape population immunity from prior infection or vaccination. These factors vary among countries within the Southern Hemisphere and certainly vary between the Southern and Northern Hemispheres. For example, activity in Australia during recent seasons has not reflected influenza virus activity in the subsequent U.S. season. Changes to the Southern Hemisphere vaccine composition, therefore, might not be a good predictor of the upcoming U.S. influenza season. Although Australia experienced an early start to its 2019 season with influenza A(H1N1)pdm09 viruses circulating initially and A(H3N2) virus eventually predominating (*2*), influenza is unpredictable, and circumstances can change very quickly. Analysis of surveillance and laboratory data to date continues to support the appropriateness of the Northern Hemisphere vaccine viruses used in production of influenza vaccines for the upcoming U.S. season.

Except for one influenza A(H1N1)pdm09 virus and one influenza B virus, all influenza viruses tested remained susceptible to oseltamivir, peramivir, and zanamivir, and only one virus contained a genetic mutation that has previously been associated with reduced susceptibility to baloxavir. Influenza antiviral medications are a valuable adjunct to annual influenza vaccination, and early treatment with influenza antiviral medication, especially within 48 hours of symptom onset, is recommended for patients with confirmed or suspected influenza who 1) have severe, complicated, or progressive illness; 2) require hospitalization; or 3) are at high risk for influenza-related complications^§§^ (*3*). Early treatment has been shown to decrease time to symptom improvement (*4*–*7*) and to reduce secondary complications associated with influenza (*8*,*9*). Health care providers should not delay treatment until test results become available because treatment is most effective when given early in the illness. Additional information regarding influenza viruses, influenza surveillance, influenza vaccines, influenza antiviral medications, and novel influenza A virus infections in humans is available at https://www.cdc.gov/flu.

**Summary**

**What is already known about this topic?**

Although influenza activity is typically low in the United States during the summer months, CDC collects, compiles, and analyzes data to monitor influenza activity throughout the year.

**What is added by this report?**

In the United States, influenza activity remained low with cocirculation of influenza A and influenza B viruses. Influenza viruses circulated widely in the Southern Hemisphere, with A(H3) viruses predominating in most regions, although influenza A(H1N1)pdm09 and influenza B/Victoria viruses predominated in several countries.

**What are the implications for public health practice?**

Receiving a seasonal influenza vaccine each year remains the best way to protect against seasonal influenza and its potentially severe consequences.

## Footnotes

* Data reported as of October 4, 2019.

† The CDC influenza surveillance system collects five categories of information from eight data sources: 1) viral surveillance (U.S. World Health Organization collaborating laboratories, the National Respiratory and Enteric Virus Surveillance System, and novel influenza A virus case reporting); 2) outpatient illness surveillance (U.S. Outpatient Influenza-Like Illness Surveillance Network); 3) mortality (the National Center for Health Statistics Mortality Surveillance System and influenza-associated pediatric mortality reports); 4) hospitalizations (FluSurv-NET, which includes the Emerging Infections Program and surveillance in three additional states); and 5) summary of the geographic spread of influenza (state and territorial epidemiologist reports). https://www.cdc.gov/flu/weekly/fluactivitysurv.htm.

§ Influenza viruses that circulate in swine are called swine influenza viruses when isolated from swine but are called variant influenza viruses when isolated from humans. Seasonal influenza viruses that circulate worldwide in the human population have important antigenic and genetic differences form influenza viruses circulating in swine. https://www.cdc.gov/flu/swineflu/variant/preventspreadfactsheet.htm.

¶ In temperate climates, the onset and peak of influenza activity might vary substantially from one influenza season to the next, but generally begins to increase in the late fall. In the Northern Hemisphere’s temperate regions, annual epidemics of influenza typically occur during October–February, but the peak of influenza activity can occur as late as April or May. In temperate regions of the Southern Hemisphere, influenza activity typically peaks during May–August. Although temperate regions of the world experience a seasonal peak in influenza activity, influenza viruses can be isolated year-round. The timing of seasonal peaks in influenza activity in tropical and subtropical countries varies by region. Multiple peaks of activity during the same year have been observed in some areas, and influenza infection can occur year-round.

** A virus is considered similar to a vaccine virus if it is well inhibited by ferret antisera raised against the cell culture– or egg culture–propagated reference virus representing the appropriate vaccine component for the specified season and hemisphere. The 2019–20 Northern Hemisphere vaccine components were A(H1N1)pdm09 subtype, A/Brisbane/02/2018-like (genetic group 6B.1A); A(H3N2) subtype, A/Kansas/14/2017-like (genetic group 3C.3a); B/Yamagata lineage, B/Phuket/3073/2013-like; and B/Victoria lineage, B/Colorado/06/2017-like (V1A.1) viruses.

†† https://www.who.int/influenza/vaccines/virus/recommendations/2020_south/en/.

§§ Persons at high risk include 1) children aged <5 years; 2) adults aged ≥65 years; 3) persons with chronic pulmonary conditions (including asthma), cardiovascular disease (except hypertension alone), renal, hepatic, hematologic (including sickle cell) disease, metabolic disorders (including diabetes mellitus), or neurologic and neurodevelopmental conditions (including disorders of the brain, spinal cord, peripheral nerves, and muscles, such as cerebral palsy, epilepsy [seizure disorders], stroke, intellectual disability [mental retardation], moderate to severe developmental delay, muscular dystrophy, or spinal cord injury); 4) persons with immunosuppression, including that caused by medications or by human immunodeficiency virus infection; 5) women who are pregnant or postpartum (within 2 weeks after delivery); 6) persons aged ≤18 years who are receiving long-term aspirin therapy; 7) American Indians/Alaska Natives; 8) persons with extreme obesity (i.e., body mass index ≥40); and 9) residents of nursing homes and other chronic care facilities.

## References

[1] Grohskopf LA, Alyanak E, Broder KR, Walter EB, Fry AM, Jernigan DB. Prevention and control of seasonal influenza with vaccines: recommendations of the Advisory Committee on Immunization Practices—United States, 2019–20 influenza season. MMWR Recomm Rep 2019;68(No. RR-3). 10.15585/mmwr.rr6803a1 31441906 PMC6713402

[2] Barr IG, Deng YM, Grau ML, Intense interseasonal influenza outbreaks, Australia, 2018/19. Euro Surveill 2019;24:1900421. 10.2807/1560-7917.ES.2019.24.33.1900421 31431210 PMC6702793

[3] Fiore AE, Fry A, Shay D, Gubareva L, Bresee JS, Uyeki TM. Antiviral agents for the treatment and chemoprophylaxis of influenza—recommendations of the Advisory Committee on Immunization Practices (ACIP). MMWR Recomm Rep 2011;60(No. RR-1). 21248682

[4] Hedrick JA, Barzilai A, Behre U, Zanamivir for treatment of symptomatic influenza A and B infection in children five to twelve years of age: a randomized controlled trial. Pediatr Infect Dis J 2000;19:410–7. 10.1097/00006454-200005000-00005 10819336

[5] Heinonen S, Silvennoinen H, Lehtinen P, Early oseltamivir treatment of influenza in children 1–3 years of age: a randomized controlled trial. Clin Infect Dis 2010;51:887–94. 10.1086/656408 20815736

[6] Nicholson KG, Aoki FY, Osterhaus AD, ; Neuraminidase Inhibitor Flu Treatment Investigator Group. Efficacy and safety of oseltamivir in treatment of acute influenza: a randomised controlled trial. Lancet 2000;355:1845–50. 10.1016/S0140-6736(00)02288-1 10866439

[7] Treanor JJ, Hayden FG, Vrooman PS, ; US Oral Neuraminidase Study Group. Efficacy and safety of the oral neuraminidase inhibitor oseltamivir in treating acute influenza: a randomized controlled trial. JAMA 2000;283:1016–24. 10.1001/jama.283.8.1016 10697061

[8] Hernán MA, Lipsitch M. Oseltamivir and risk of lower respiratory tract complications in patients with flu symptoms: a meta-analysis of eleven randomized clinical trials. Clin Infect Dis 2011;53:277–9. 10.1093/cid/cir400 21677258 PMC3137795

[9] Lipsitch M, Hernán MA. Oseltamivir effect on antibiotic-treated lower respiratory tract complications in virologically positive randomized trial participants. Clin Infect Dis 2013;57:1368–9. 10.1093/cid/cit481 23883518 PMC3792722

